# Metastatic pancreatic ductal adenocarcinoma followed by a fatal diffuse large B-cell lymphoma: A rare case report and literature review

**DOI:** 10.1097/MD.0000000000033217

**Published:** 2023-03-24

**Authors:** Atsushi Yamaguchi, Naohiro Kato, Shuhei Sugata, Takuro Hamada, Nao Furuya, Takeshi Mizumoto, Yuzuru Tamaru, Ryusaku Kusunoki, Toshio Kuwai, Hirotaka Kouno, Miki Kido, Takuo Ito, Kazuya Kuraoka, Hiroshi Kohno

**Affiliations:** a Department of Gastroenterology, National Hospital Organization Kure Medical Center and Chugoku Cancer Center, Kure, Japan; b Department of Hematology, National Hospital Organization Kure Medical Center and Chugoku Cancer Center, Kure, Japan; c Department of Pathology, National Hospital Organization Kure Medical Center and Chugoku Cancer Center, Kure, Japan.

**Keywords:** case report, double cancer, lymphoma, multiple primary malignancies, pancreatic cancer, pancreatic ductal adenocarcinoma, polyoncosis

## Abstract

Recently, the incidence of polyoncosis has been increasing due to advancements in treatment, such as antitumor therapy, which led to a prolonged survival. However, few patients with metastatic pancreatic ductal adenocarcinoma (PDAC) develop second tumors, which render a poor prognosis. We report a rare case of PDAC, which is metachronous with a fatal malignant lymphoma (ML).

**Patient concerns:**

A 68-year-old woman who had been monitored due to liver cirrhosis secondary to hepatitis C virus infection presented with a 10-mm pancreatic head cancer with lung metastasis and had started an anticancer therapy with gemcitabine. Approximately 18 months after diagnosis, lymphadenopathies around the pancreas were noted, which eventually spread to the entire body over time.

**Diagnosis:**

Diffuse large B-cell lymphoma was diagnosed using biopsies from cervical lymph nodes.

**Interventions and outcomes:**

The patient started a gemcitabine + rituximab regimen; however, the patient died from cachexia-associated lymphoma progression, not PDAC.

**Lessons:**

ML should be considered when intra-abdominal lymphadenopathies are detected in patients with pancreatic cancer, and ML should be differentiated from lymph node metastasis of pancreatic cancer.

## 1. Introduction

Recently, patients with multiple primary malignancies (e.g., multiple primary tumors, double cancer, or polyoncosis) have been increasing due to advancements in antitumor therapy, thereby prolonging life. However, a few patients with metastatic pancreatic ductal adenocarcinoma (PDAC) develop second tumors after PDAC, which has a poor prognosis. We report a case of patient with PDAC who developed fatal malignant lymphoma (ML) originating from the para-pancreatic lymph nodes (LNs).

## 2. Case report

**Figure 1.**
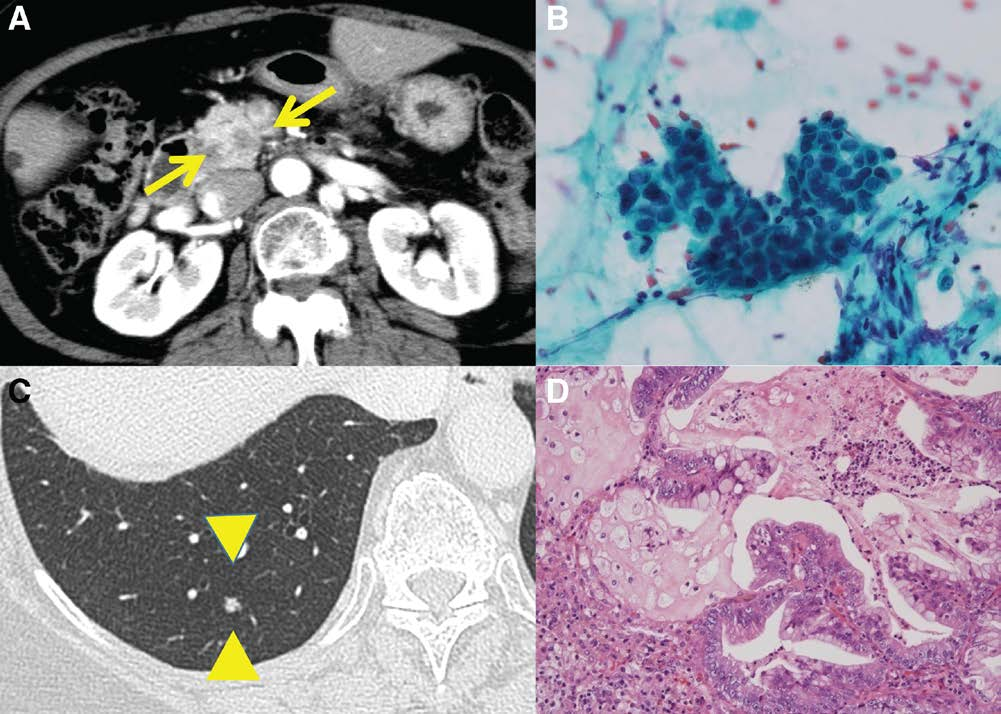
Abdominal computed tomography scan revealed a 10-mm hypodense mass in the pancreatic head (A), pancreatic juice was taken during endoscopic retrograde pancreatography, and cytological examination revealed a moderately differentiated adenocarcinoma (B). A 4-mm right lower lung lobe mass was noted (C) and confirmed to be a metastatic tumor from pancreatic cancer via (D) laparoscopic partial lung excision, where the histologic picture was similar to a pancreatic tumor.

**Figure 2.**
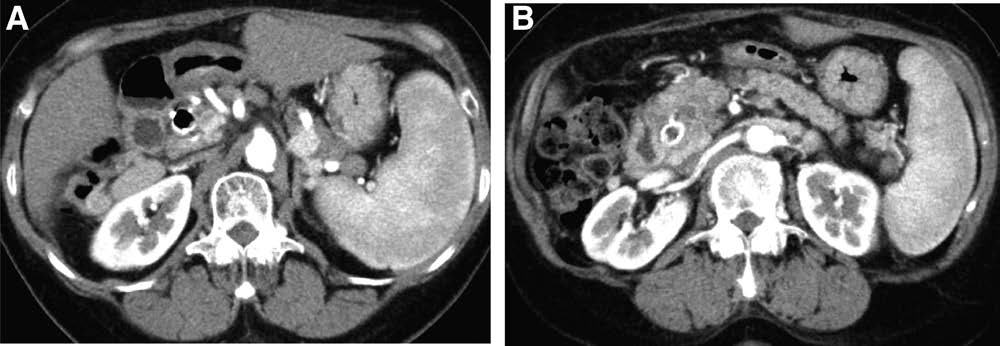
At 18 months after the diagnosis, a computed tomography scan revealed lymphadenopathies around the pancreatic body (A) but no remarkable change in the pancreatic tumor size (B).

**Figure 3.**
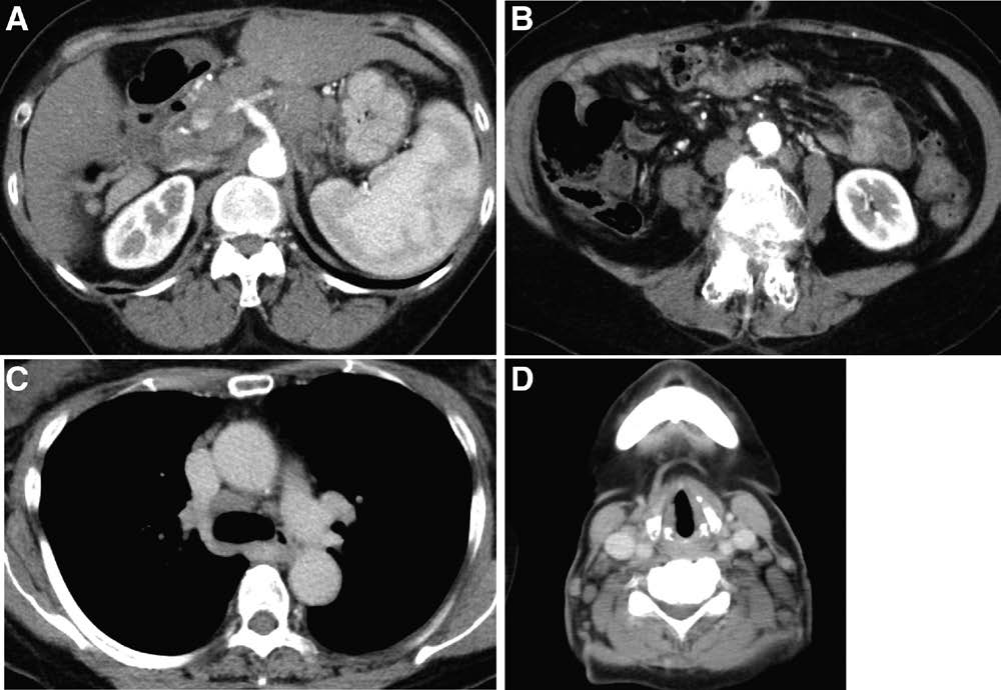
At 21 months, lymphadenopathies were noted around the abdominal aorta (A, B) and mediastinum (C). At 23 months, cervical lymphadenopathies were noted (D).

This is a case of a 68-year-old woman who was on anti-viral therapy for hepatitis C virus (HCV) infection and medically cured in 2006. The patient underwent computed tomography (CT) or abdominal ultrasonography once every 6 months for hepatocellular carcinoma surveillance. In 2012, an abdominal CT scan revealed a 10-mm hypodense mass (Fig. 1A) in the pancreatic head, and further examinations were performed. Although the patient had been cured of HCV but had already developed liver cirrhosis during that period. The patient had no history of chronic pancreatitis. The patient did not smoke or drink alcohol. The patient had no family history of cancer. Physical examination results on admission were as follows: height, 156 cm; weight, 50 kg. Abdomen was soft and flat with no palpable masses. Relevant laboratory data include glutamic oxaloacetic transaminase 17 IU/L, glutamic pyruvic transaminase 9 IU/L, and carcinoembryonic antigen 10.2 ng/mL. Carbohydrate antigen 19-9, soluble interleukin-2 receptor, and pancreatic enzymes were within the normal range. HCV antibody and hepatitis B virus c antibody were positive. Pancreatic juice was taken during endoscopic retrograde pancreatography (Fig. 1B); cytological examination revealed a moderately differentiated adenocarcinoma, and the patient was diagnosed with pancreatic carcinoma. In addition, the patient had a 4-mm mass in the right lower lung lobe (Fig. 1C). A laparoscopic partial lung excision was performed and the species showed a histologic picture similar to a pancreatic tumor (Fig. 1D), positive for CK7/20 and negative for TTF-1. Thus, she was determined as a metastatic tumor from pancreatic cancer (PC). Antitumor therapy with gemcitabine (GEM) was initiated. During the 9-month follow-up visit, GEM regimen was revised to tegafur, gimeracil, and oteracil potassium due to the new-onset of lung metastasis. At 14 months, the patient developed obstructive jaundice and underwent biliary metallic stent placement. The patient also had massive ascites from liver cirrhosis and biliary obstruction at that time. Although ascites resolved naturally in a few months, but the patient refused anticancer therapy and was unavailable for follow-up. At 18 months, the patient sought a consultation with our hospital. A CT scan was performed, which revealed lymphadenopathies around the pancreas (Fig. 2A) but with no remarkable change in the pancreatic tumor size (Fig. 2B). At 21 months, lymphadenopathies were noted at the abdominal aorta (Fig. 3A and B) and in the mediastinum (Fig. 3C).

**Figure 4.**
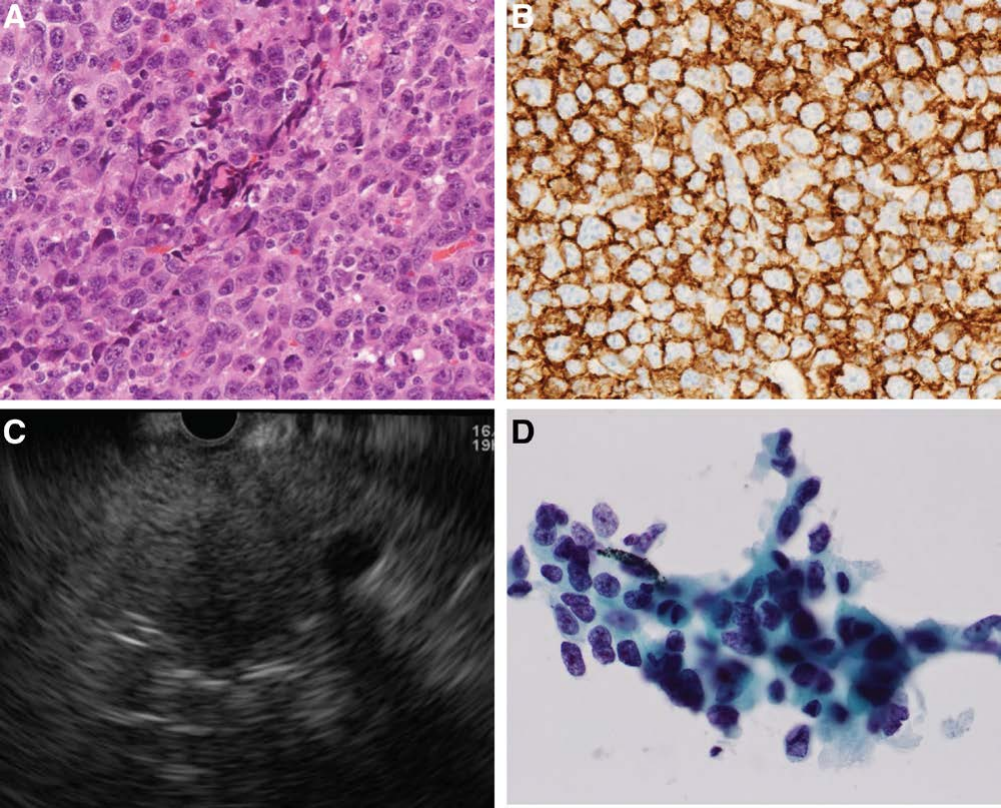
A cervical lymph node biopsy was conducted, and diffuse large B-cell lymphoma was diagnosed (A: hematoxylin and eosin stain, B: positive for CD20). The pancreatic tumor was reconfirmed as adenocarcinoma (C, D) using fine-needle aspiration cytology via endoscopic ultrasonography guidance.

Furthermore, at 23 months, cervical (Fig. 3D), pelvic, and femoral inguinal lymphadenopathies were observed, and ML was suspected. After that, we performed a cervical LN biopsy, and diffuse large B-cell lymphoma (DLBCL) was confirmed (Fig. 4A and B). In addition, a fine-needle aspiration cytology was performed via endoscopic ultrasonography guidance, and the pancreatic tumor was reconfirmed as adenocarcinoma (Fig. 4C and D). The patient opted for a weaker anticancer therapy, and the GEM + rituximab regimen was selected, not cyclophosphamide, hydroxydaunorubicin, oncovin, and prednisone + rituximab. PC did not progress; however, lymphoma progressed, and the patient developed cancer cachexia and died 32 months after diagnosis of PC (9 month after ML). Autopsy was performed and the cause of death was determined to be cachexia and progression of ML. Moreover, the primary PC was approximately 20 mm, and lung and liver metastases were deemed less severe.

## 3. Discussion

We encountered a rare case of a patient with metastatic PC who developed DLBCL and died during the therapeutic course.

Multiple primary malignancies are defined as two or more primary malignant tumors diagnosed in one individual, also referred to as multiple primary tumors, double cancers, and polyoncosis. They are further classified as synchronous or metachronous based on the period of each cancer diagnosis.

In 1932, Warren and Gates^[1]^ reported that the frequency of polyoncosis in autopsy cases was approximately 1.8%. Moreover, cases of polyoncosis have been increasing due to advancements in antitumor therapy and prolonged survival. In Japan, many studies on polyoncosis were reported between the 1990s to the 2010s. The frequency of double cancers associated with PC was reported to be 6.4% to 20.4%^[2,3]^ in autopsy cases and 7.6% to 13.2%^[4,5]^ in clinical cases, which is deemed high compared to other tumors. In addition, tumor types that accompany PC include gastric (38.8%–42.3%), colon, breast, and thyroid cancers; however, this distribution was not characteristic of PDAC.^[4–6]^ In polyoncosis associated with PC, few patients were reported to have developed other tumors, but most died from PC,^[3–6]^ probably attributed to its poor prognosis.

Concurrent PDAC and ML are rare, and both diseases have poor prognoses. A study reported that 630 cases of non-Hodgkin’s lymphoma accompanied 31 other cancers, three of which were PC.^[7]^ In contrast, other reports revealed that ML did not accompany PC. Thus, concurrent ML and PC are very rare. We searched PCs whose ML existed simultaneously during PC diagnosis and developed after the initiation of PC therapy. We collected 2 cases^[8]^ from PubMed using the keywords: “pancreatic cancer” and “malignant lymphoma,” as well as 2 cases^[9,10]^ from *Igaku tyuo zassi* (Japan) using the keywords: “suigann” and “akuseirinnpasyu”; “tyoufukusyuyou” and “akuseirinnpasyu”; and “tyoufukusyuyou” and “suigann.” We reviewed these 4 cases, including our case (Table 1). In these 5 cases, there were 4 patients with synchronous tumors where PC and ML were found simultaneously. Our case was metachronous, and ML followed PC. Three cases were prediagnosed as PC plus LN metastasis and were diagnosed with concurrent PC plus ML after the surgeries. One patient had PC in the pancreatic head and multiple LNs around the peri-pancreatic area; however, the initial diagnosis was LN metastasis from PC. ML was diagnosed 5 months later, and the LNs spread throughout the body. This case was the same as ours, where ML was diagnosed late.

**Table 1:** Five cases of PDAC accompanied with malignant lymphoma in concurrent or after PDAC.

Author	Reported year	Age	Sex	Synchronous/metachronous	Feature of PDAC						Feature of ML					Prognosis	Ref.
					L	TS	N	M	H-type	Therapy	H-type	Localization (at PDAC diagnosis)	Localization (at ML diagnosis)	Methods of diagnosis	Therapy		
Koyama S, et al	1983	75	M	Synchronous (concurrent)	H	40	-	-	mod	Fluorouracil + Mitomycin C	DLBCL	LN (Peri-pancreas)	LN (Peri-pancreas, Retroperitoneum, Mesentery, Hilar, Armpit), Organs (Pancreas, Stomach, Heart, Bone, etc)	Biopsy from subcutaneous nodules	-	Death from ML in 6.5 M after PDAC diagnosis andin 0.5 M after ML diagnosis	^[8]^
Imai H, et al	1991	62	M	Synchronous (concurrent)	H	20	-	-	mod	TP	DLBCL	LN (Peri-pancreas, around SMA)	Same as left	Surgery for biliary bypass	Cyclophosphamide + mercaptopurineafter PD	Relapse-free (both PDCA and ML) survival in 19 M	^[9]^
Lai JM, et al	2011	70	F	Synchronous (concurrent)	H	N.A.	-	-	IPMC (N.A)	PD->GEM	follicular	LN (Aorta-caval, retroportal, portal hepatic)	Same as left	Surgery for PDAC	-	Relapse-free (both PDCA and ML) survival in 12 M	^[10]^
Lai JM, et al	2011	78	M	Synchronous (concurrent)	H	30	-	-	por	PD->GEM	lymphocytic	LN (Peri-pancreatic, omental, retro-portal, hepatic artery)	Same as left	Surgery for PDAC	-	Relapse-free (both PDCA and ML) survival in 12 M	^[10]^
Our case		68	F	Metachronous (18 mo after PDAC)	H	10	N.A.	Lung	mod ~ por	GEM-> S1	DLBCL	-	LN (Peri-pancreas, Retroperitoneum, Mediastinum, Inguinal, Mesentery, Armpit, etc)	Biopsy from cervical nodules	GEM + rituximub	Death from ML in 32 M after PDAC diagnosis andin 9 M after ML diagnosis	

- = none, DLBCL = diffuse large B cell lymphoma, F = female, GEM = gemcitabine, H = head, H-type = histological type, IPMN = intraductal papillary mucinous neoplasm, L = localization of PDAC, LN = lymph node, M = distant metastasis, M = male, ML = malignant lymphoma, mod = moderately differentiated, N = lymph node metastasis, N.A = not available, PD = pancreatoduodenectomy, PDAC = pancreatic ductal adenocarcinoma, por = poorly differentiated, Ref. = reference number, S1 = tegafur, Gimeracil, Oteracil Potassium, SMA = superior mesenteric artery, TP = total pancreatectomy, TS = tumor size.

All MLs were included in non-Hodgkin’s lymphoma and classified in DLBCLs, follicular lymphoma, and lymphocytic lymphoma. All PCs were located in the pancreatic head. Notably, the primary sites of ML were all in the upper abdomen (especially the peri-pancreas), whereas non-Hodgkin’s lymphoma often develops in various LNs and organs. The development of ML is attributed to multiple factors (e.g., EB virus infection,^[11,12]^ HCV infection,^[11,13]^ helicobacter pylori infection,^[11,14]^ family history,^[11]^ autoimmune diseases,^[11]^ radiation,^[11,15]^ some inflammatory diseases, and aging^[11]^) and immune suppression.^[11,16,17]^ In our patient, immune suppression secondary to HCV infection plus liver cirrhosis, PC, and antitumor therapy might had induced the development of ML. In reference to the 5 cases mentioned, we speculated that PC could induce ML in the upper abdominal LNs.

When abdominal malignant tumors accompanied by lymphadenopathies occur, a concurrent lymphoma should be differentiated from LN metastasis. The following imaging characteristics are used to differentiate ML from LN metastasis of PC: few LN metastases in the para-aortic area and under the renal vein in PC; few invasions and obstruction of portal vein whereas exclusion; few diffuse lymphadenopathies in the abdomen in PC; irregular LN borders and unclear boundaries in PC; lymphadenopathies are larger in ML than in PC; and sandwiching of intestinal membrane vessels by multiple LNs (sandwich sign) is characteristic of ML.^[18,19]^ During the development of lymphadenopathies in our patient, CT imaging already revealed images suggestive of ML. An ML that is concurrent with PC should have been suspected.

Another dilemma, in this case, is the selection of therapy. For lymphomas concurrent with other cancers, therapy for ML is prioritized, followed by therapy for other cancers, considering the higher malignant potential of ML. Although metastatic PC is also highly malignant comparable to ML, in our case, the speed of growth of LNs was faster than the PC-associated lesions; thus, ML therapy was selected. The GEM plus rituximab regimen was not effective, and we speculate that the rituximab plus cyclophosphamide, hydroxydaunorubicin, oncovin, and prednisone regimen could have been effective for the patient. In the literature research, patients with DLBCL had a poorer prognosis than those with follicular and lymphocytic lymphomas, with a relatively better prognosis even without therapy. Malignant potential and growth speed of ML should be considered in therapy selection.

## 4. Conclusion

We encountered a case of metastatic PC followed by fatal ML. In patients with PC who present with intra-abdominal lymphadenopathies (especially in the peri-pancreatic area), ML should be considered. Furthermore, ML should be differentiated from LN metastasis of PC using imaging characteristics and fine-needle aspiration biopsy via endoscopic ultrasonography guidance.

## Author contributions

**Conceptualization:** Atsushi Yamaguchi.

**Data curation:** Naohiro Kato, Shuhei Sugata, Takuro Hamada, Nao Furuya.

**Writing – original draft:** Atsushi Yamaguchi.

**Writing – review & editing:** Takeshi Mizumoto, Yuzuru Tamaru, Ryusaku Kusunoki, Toshio Kuwai, Hirotaka Kouno, Miki Kido, Takuo Ito, Kazuya Kuraoka, Hiroshi Kohno.
